# Supplementary material for: New Bioinformatic Insight into CD44: Classification of Human Variants and Structural Analysis of CD44 Targeting
Source: Int J Mol Sci. 2025 Oct 11;26(20):9886. doi: 10.3390/ijms26209886 (PMC12563948; doi:10.3390/ijms26209886)
Supplement: Supplementary file 1 [file ijms-26-09886-s001.zip › ijms-3880736-supplementary.pdf]

New bioinformatic insight into CD44: classification of human variants and structural analysis of CD44 targeting

Wiktoria A. Gerlicz, Aleksandra Olczak, Aneta M. Białkowska and Aleksandra Twarda-Clapa*

Institute of Molecular and Industrial Biotechnology, Faculty of Biotechnology and Food Sciences, Lodz University of Technology, 90-537 Lodz, Poland.; * Correspondence: aleksandra.twarda-clapa@p.lodz.pl

Supplementary Materials

A

B

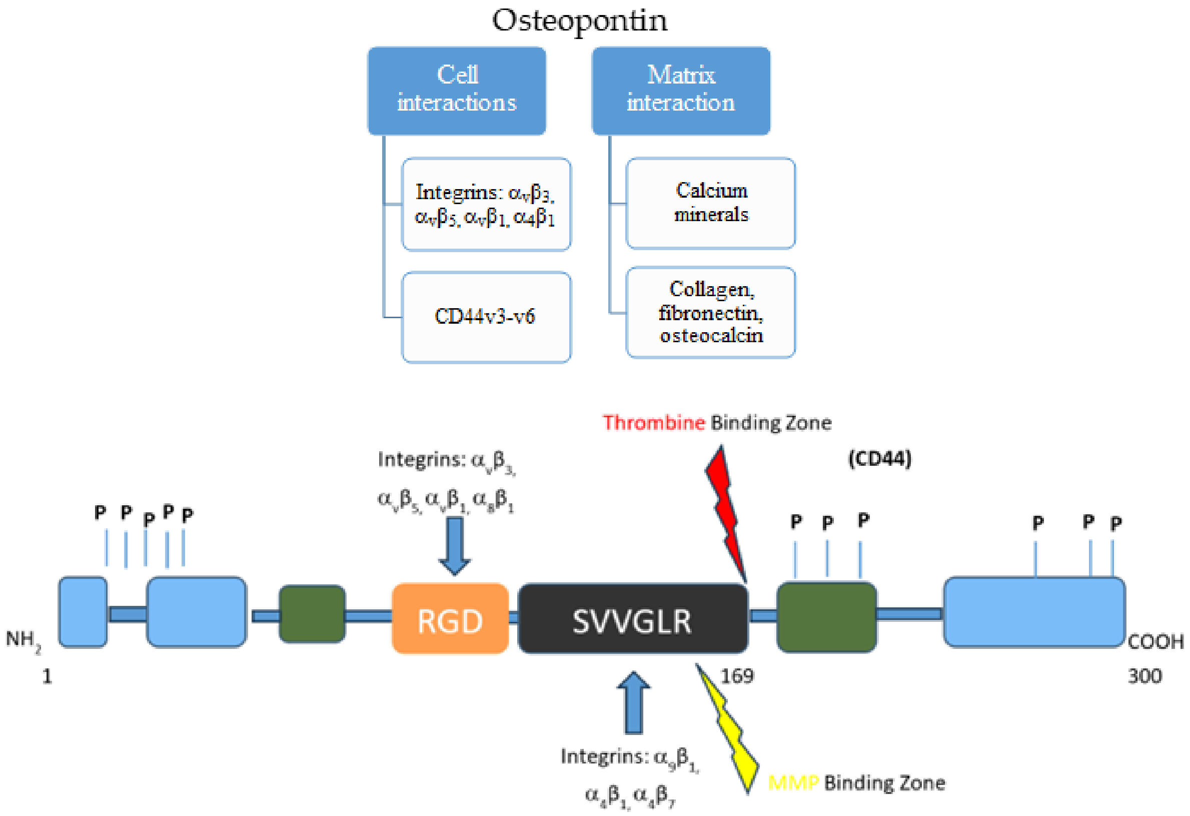

C

Inflammation	Cancer	Diabetes	Wound healing	Nephrolithiasis	Biomineralization
Increase in macrophage and T cel count	Increase in cell migration	Increase in insulin resistance	Increase in macrophage infiltration and TGF-β secretion	Prevention of Caox crystal formation and accumulation	Modulation of osteoclast function

Figure S1. Structure and functions of osteopontin (OPN). A. Cell and matrix interactions of OPN; B. Structure of OPN; C. Schematic representation of the effects of OPN on health. Based on [28].

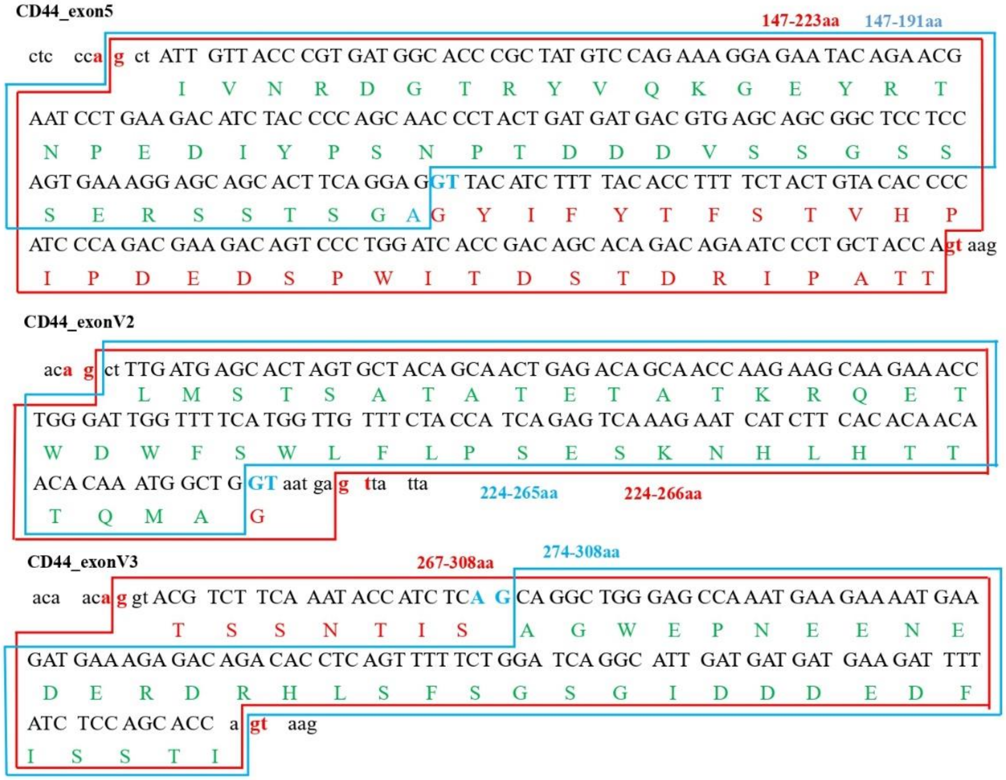

Figure S2. CD44 gene (GRCh38.p14) fragments coding exons 5, 6 (V2) and 7 (V3), and the 5-8bp noncoding nucleotides at each end of the exons. In green, the protein sequence consistent for both the full form and microexon is shown, with full-form amino acids indicated in red and microexon specific ones indicates in blue. In red frame, the full form of the exon is shown with its conventional splice sites shown in red. Similarly, in the blue frame the microexon variant is shown, with the alternative donors/acceptors given in blue.

Table S1. Exon map according to Screaton, et al., 1992 [47].

Exon number	Amino acid positions	Amino acid sequence
1	1-23	MDKFWWHAAWGLCLVPLSLAQID
2	24-78	LNITCRFAGVFHVEKNGRYSISRTEAADLCKAFNSTLPTMAQMEKALSIGFETCR
3	79-123	YGFIEGHVVIPRIHPNSICAANNTGVYILTSNTSQYDTYCFNASA
4	124-146	PPEEDCTSVTDLPNAFDGPITIT
5	147-223	IVNRDGTRYVQKGEYRTNPEDIYPSNPTDDDVSSGSSSERSSTSGGYIFYTFSTVHPIPDEDSPWIT DSTDRIPATT
6b (V2)	224-266	LMSTSATATETATKRQETWDWFSWLFLPSESKNHLHTTTQMAG
7 (V3)	267-308	TSSNTISAGWEPNEENEDERDRHLSFSGSGIDDDEDFISSTI
8 (V4)	309-346	STTPRAFDHTKQNQDWTQWNPSHSNPEVLLQTTTRMTD
9 (V5)	347-385	VDRNGTTAYEGNWNPEAHPPLIHHEHHEEEETPHSTSTI
10 (V6)	386-428	QATPSSTTEETATQKEQWFGNRWHEGYRQTPKEDSHSTTGTAA
11 (V7)	429-472	ASAHTSHPMQGRTTPSPEDSSWTDFFNPISHPMGRGHQAGRRMD
12 (V8)	473-506	MDSSHSITLQPTANPNTGLVEDLDRTGPLSMTTQ
13 (V9)	507-536	QSNSQSFSTSHEGLEEDKDHPTTSTLTSSN
14 (V10)	537-604	RNDVTGGRRDPNHSEGSTTLLEGYTSHYPHTKESRTFIPVTSAKTGSFGVTAVTVGDSNSNVNRS LSG
15	605-625	DQDTFHPSGGSHTTHGSESDG
16	626-649	HSHGSQEGGANTTSGPIRTPQIPE
17	650-675	WLIILASLLALALILAVCIAVNSRRR
18	-	„characteristic 3’UTR region”
19	676-742	CGQKKKLVINSGNGAVEDRKPSGLNGEASKSQEMVHLVNKESSETPDQFMTADETRNLQNVD MKIGV

Table S2. Complete list of known and proposed CD44 isoforms grouped by the exons they contain, with minor additional variations listed for each sequence within a group. Additionally, the first reference found for each variant is provided. Unpublished references are marked with the (*) symbol.

Exon(s) spliced out	*Isoform name(s)*	*Length [aa]*	*Additional differences*	*NCBI accession*	*UniProt accession*	*First reference*
N/A (Full-length form)	CD44v2-10 canonical NCBI 1 UniProt 1	742		NP_000601.3	P16070-1	Screaton, et al., 1992; Shtivelman & Bishop, 1991 [47,52]
	unnamed isoform	742	p.K417R p.I479T	AAB13628.1 KAI4070736.1		Screaton, et al., 1992; Shtivelman & Bishop, 1991 [47,52]
	unnamed isoform	742	p.S109Y p.T241A p.K417R p.I479T p.D494N	CAB61878.1		Günthert, 1993 [53]
	CRA_d	742	p.K417R	EAW68148.1 EAW68147.1 KAI2559389.1		Venter, et al., 2001 [59]
	NCBI X2	742	p.345_346insA p.K417R p.A428del p.I479T	XP_054226535.1		T2T-CHM13v2.0 annotation
	NCBI X2 (alternate)	742	p.345_346insA p.A428del	XP_005253289.1		GRCh38p14 annotation
	NCBI 9	743	p.345_346insA	NP_001427253.1		Cobley, 2012 (*) [55] annotation
	NCBI 10	741	p.A428del	NP_001427254.1		Cobley, 2012 (*) [55] annotation
**Exons 2-19** p.23_742delins GVGRRKS	CD44SP UniProt 2	29		AAB27917.1	P16070-2	Tanabe, et al., 1993 [57]
**Exons 3-19** p.78_742delinsSST	unnamed isoform	80		AAH52287.1 KAI2559398.1 KAI4070737.1	-	Strausberg, et al., 2002 (Mammalian Gene Collection Programme Team) [54]
**Exons 3-19** p.78_742delinsSLHCS QQSKKVWAEEKAS DQQWQWSCGGQK CGGQKAKWTQRRG QQVSGNGAFGEQG VVRNSRPVYDS	CD44sol CD44RC, CRA_g NCBI 5 UniProt 19	139		NP_001001392.1 AAC70782.1 EAW68152.1 KAI2559396.1 KAI4070741.1	P16070-19	Chiu, et al., 1998 (*) [58]
**Part of exon 5** p.192_223delinsA	UniProt 3	711	-		P16070-3	Unknown
	NCBI 11	712	p.345_346insA	NP_001427255.1		Cobley, 2012 (*) [55] annotation

Exon(s) spliced out	*Isoform name(s)*	*Length [aa]*	*Additional differences*	*NCBI accession*	*UniProt accession*	*First reference*
**Part of exon 5** p.192_223delinsA **Exon V6** p.385_428delinsT	unnamed isoform	668	p.I479T	AAB13626.1		Screaton, et al., 1992; Shtivelman & Bishop, 1991 [47,52]
	CRA_h UniProt 16	668		EAW68153.1	P16070-16	Venter, et al., 2001 [59]
**Exon V2** p.223_266delinsS	CD44v3-10 epidermal epican NCBI 2 UniProt 4	699		NP_001001389.1	P16070-4	Kugelman, et al., 1992 [60]
	epican	699	p.E410V p.I479T	CAA47271.1		Kugelman, et al., 1992 [60]
	CRA_f	699	p.K417R	EAW68150.1 KAI2559390.1		Strausberg, et al., 2002 [54]
	unnamed isoform	699	K417R p.I479T	AAH04372.1 KAI4070734.1		Strausberg, et al., 2002 [54]
	NCBI 12	700	p.345_346insA	NP_001427256.1		Cobley, 2012 (*) [55] annotation
	NCBI 13	699	p.345_346insA p.A428del	NP_0001427257.1		Cobley, 2012 (*) [55] annotation
**Exon V2** p.223_266delinsS **Exons V4-V7** p.308_472delinsN **Exons V10** p.536_604delinsR	NCBI 34	467		NP_001427278.1		Cobley, 2012 (*) [55] annotation
**Exon V2** p.223_266delinsS **Exons V6-V7** p.385_472delinsN	NCBI 25	613	p.345_346insA	NP_001427269.1		Cobley, 2012 (*) [55] annotation
**Exon V2** p.223_266delinsS **Exon V7** p.428_472delinsD	NCBI 22	656	p.345_346insA	NP_001427266.1		Cobley, 2012 (*) [55] annotation
**Exon V2** p.223_266delinsS **Exons V7-19** p.428_742delinsGDCG SMAWVKKYFSFIFL	NCBI 36	403	p.345_346insA	NP_001427280.1	-	Cobley, 2012 (*) [55] annotation
	NCBI 37	402		NP_001427281.1	-	Cobley, 2012 (*) [55] annotation
**Exon V2** p.223_266delinsS **Exon V10** p.536_604delinsR	NCBI 23	631		NP_001427267.1		Cobley, 2012 (*) [55] annotation
**Exons V2-V3** p.223_308delinsI	NCBI 20	658	p.345_346insA	NP_001427264.1		Cobley, 2012 (*) [55] annotation
	NCBI 21	657		NP_001427265.1		Cobley, 2012 (*) [55] annotation

Exon(s) spliced out	*Isoform name(s)*	*Length [aa]*	*Additional differences*	*NCBI accession*	*UniProt accession*	*First reference*
	NCBI X11	656	p.K417R<br>p.A428del<br>p.I479T	XP_054226544.1		T2T-CHM13v2.0 annotation
	NCBI X11 (alternate)	656	p.A428del	P_011518787.1		GRCh38p14 annotation
**Exons V2-V3**<br>p.223_308delinsI<br>**Exon V7**<br>p.428_472delinsD	NCBI 24	614	p.345_346insA	NP_001427268.1		Cobley, 2012 (*) [55] annotation
**Exons V2-V4**<br>p.223_385delinsI	NCBI 26	580		NP_001427270.1		Cobley, 2012 (*) [55] annotation
	NCBI 27	579	p.A428del	NP_001427271.1		Cobley, 2012 (*) [55] annotation
**Exon V2**<br>p.223_266delinsS<br>**Exons V4-V7**<br>p.308_472delinsN	NCBI 31<br>CRA_j	535		NP_001427275.1<br>EAW68155.1		Venter, et al., 2001 [59]
**Exons V2-V5**<br>p.223_385delinsI<br>**Exon V7**<br>p.428_472delinsD	NCBI 30	536		NP_001427274.1	-	Cobley, 2012 (*) [55] annotation
**Exons V2-V5**<br>p.223_385delinsI<br>**Exon V7**<br>p.428_472delinsD<br>**Exons V10**<br>p.536_604delinsR	NCBI 33	468		NP_001427277.1		Cobley, 2012 (*) [55] annotation
**Exons V2-V5**<br>p.223_385delinsI<br>**Exons V10**<br>p.536_604delinsR	NCBI 32	512		NP_001427276.1		Cobley, 2012 (*) [55] annotation
**Exons V2-V6**<br>p.223_428delinsT	NCBI 29	537		NP_001427273.1	-	Cobley, 2012 (*) [55] annotation
**Exons V2-V7**<br>p.223_472delinsN	CD44v8-10<br>epithelial<br>keratinocyte<br>CD44E<br>CRA_e<br>NCBI 3<br>UniProt 10	493		**NP_001001390.1**<br>EAW68151.1<br>EAW68149.1<br>KAI2559391.1	P16070-10	Stamenkovic, et al., 1991 [61]
	CD44R1	493	p.I479T	CAA40133.1<br>AAB13627.1<br>KAI4070739.1	-	Dougherty, et al,. 1991 [62]
**Exons V2-V7**<br>p.223_472delinsN<br>**Exons V9-V10**<br>p.506_604delinsR	UniProt 14	396		-	P16070-14	Tanabe, et al., 1993 [57]
	CRA_i	396		EAW68154.1	-	Venter, et al., 2001 [59]
	CD44R5	395	p.I479T	AAB27919.1	-	Tanabe, et al., 1993 [57]

Exon(s) spliced out	*Isoform name(s)*	*Length [aa]*	*Additional differences*	*NCBI accession*	*UniProt accession*	*First reference*
**Exons V2-V7** p.223_472delinsN **Exon V10** p.536-604delinsR	CD44R4 UniProt 13	425	p.I479T	AAB27918.2	P16070-13	Tanabe, et al., 1992 [57]
	NCBI 35 CRA_b	425		NP_001427279.1 EAW68145.1	-	Venter, et al., 2001 [59]
**Exons V2-V9** p.223_536delinsN	CD44v10 CD44R2 NCBI 6 UniProt 11	429		NP_001189484.1 KAI2559392.1 KAI4070742.1	P16070-11	Dougherty, et al., 1991 [62]
**Exons V2-V10** p.223_604delinsR	CD44s standard CDw44 reticulocyte CRA_a CD44H haematopoietic NCBI 4 UniProt 12	361		NP_001001391.1 EAW68144.1 AAB13624.1 KAI2559393.1 KAI4070735.1	P16070-12	Stamenkovic, et al., 1989 [11]
	unnamed isoform	361	p.S109Y	AAA51950.1	-	Harn et al. 1994 (*) [64]
	unnamed isoform	361	pp.S697I	AAH67348.1 AAM50041.1	-	Wiebe et al. 2002 (*) [65]
	unnamed isoform	361	p.H92Q	AXZ96474.1	-	Stettler et al. 2018 (*) [66]
**Exons V2-V10** p.223_604delinsR **Exon 19** p.675_742delinsS	CD44st "short tail" Hermes NCBI 8 UniProt 15	294		NP_001189486.1 AAB13622.1	P16070-15 H0Y5E4	Goldstein et al. 1989 [9]
**Exons V2-15** p.223_625delinsR	CD44s-exon15 NCBI 7 UniProt 18	340		NP_001189485.1 KAI2559394.1 KAI4070743.1	P16070-18	Fang et al. 2008 (*) [67]
**Part of exon V3** p.266_273delinsA	UniProt 5	734	-		P16070-5	Unknown
**Part of exon V3** p.G266_S273del **Exon V6** p.385_428delinsT	CRA_c UniProt 17	691		EAW68146.1	P16070-17	Venter, et al., 2001 [59]
	unnamed isoform	691	p.I479T	AAB13625.1		Screaton, et al., 1992; Shtivelman & Bishop, 1991 [47,52]
**Exons V4-V7** p.308_472delinsN	NCBI 28	578		NP_001427272.1		Cobley, 2012 (*) [55] annotation
**Exons V4-19** p.308_742delinsIICLFTR RIYKQHTVTKSLGF QVQRDTTDCMDGQN GAFGYPRWRAGVFK AVLPTAAASLTVLSG RSHVLNPKVFYDRM QRTLRCLPIWLN	NCBI 38	397		NP_001427282.1		Cobley, 2012 (*) [55] annotation
**Exon V6** p.385_428delinsT	UniProt 6	699			P16070-6	Unknown

Exon(s) spliced out	*Isoform name(s)*	*Length [aa]*	*Additional differences*	*NCBI accession*	*UniProt accession*	*First reference*
**Exon V7**<br>p.428_472delinsD	NCBI X6	699	p.345_346insA<br>p.K417R<br>p.I479T	XP_054226539.1		T2T-CHM13v2.0 annotation
	NCBI X6 (alternate)	699	p.345_346insA	XP_016874072.1	-	GRCh38p14 annotation
**Exon V7-19**<br>p.428_742delinsGDCGSM AWVKKYFSFIFL	NCBI X22	446	p.345_346insA<br>p.K417R	XP_054226555.1		T2T-CHM13v2.0 annotation
	NCBI X22 (alternate)	446	p.345_346insA	XP_011518789.1		GRCh38p14 annotation
**Exon V9**<br>p.506_535delinsR	UniProt 7	713	-		P16070-7	Unknown
**Exon V10**<br>p.536_604delinsR	UniProt 8	674			P16070-8	Unknown
	NCBI X7	675	p.345_346insA<br>p.K417R<br>p.I479T	XP_054226540.1		T2T-CHM13v2.0 annotation
	NCBI X7 (alternate)	675	p.345_346insA	XP_011518784.1	-	GRCh38p14 annotation
**Exon 19**<br>p.675_742delinsS	UniProt 9	675			P16070-9	Unknown
	unnamed isoform	675	p.K417R<br>p.I479T	AAB13623.1		Screaton, et al., 1992; Shtivelman & Bishop, 1991 [47,52]

Table S3. List of all partial sequences from UniProt and NCBI Protein databases, with the identical entries matched together.

Length [aa]	NCBI abbreviation	UniProt abbreviation
361	ARX77882.1	
337	CAA44602.1	
287	KAI2559400.1<br>KAI4070744.1	H0Y2P0
277		H0YCV9
277	KAI2559397.1	
277	KAI4070740.1	
269	KAI2559402.1	
269	KAI4070746.1	
268		H0YEU1
243		H0YEV3
240		H0YDW7
232	KAI2559404.1	J3KN83
232	KAI4070748.1	
229		H0YD17
206	KAI2559395.1	H0YD13
199	ARX77882.1	
195		H0YCL4
195		H0YD90
195	KAI2559406.1	
195	KAI4070750.1	
193	CAA39404.1	
193	KAI2559405.1	H0YEA1
193	KAI4070749.1	

Length [aa]	NCBI abbreviation	UniProt abbreviation
191		H0YDV8
187	KAI2559403.1<br>KAI4070747.1	
186		H0YF08
183		H0YDX6
177	AAD14389.1	
176	KAI4070745.1<br>KAI2559401.1	
128	AAD14279.2	
118		H0YES0
82	KAI2559407.1	H0YE40
82	KAI4070751.1	
80	AAH52287.1	Q86UZ1
78		E9PKC6
55	UZP80211.1	
45	BAH57528.1	
44	AAB30429.1	
42	BAH57527.1	
41	ABW75083.1	
41	BAH30705.1	
33	AAD00766.1	
31	AAD00765.1	
29	BAH57879.1	
2	BAA05813.1	

Table S4. Methods used for the preparation and sequencing of the biological isoforms of CD44. For other, not mentioned authors, methods are unknown (e.g., because the manuscript has not been published).

Reference	Method
Goldstein, et al., 1989 [9]	RNA isolated from KCA human lymphoblastic cells to create cDNA library; two subsequent screenings with anti-gp90 Abs, followed by a re-screening with the clone strongly reacting with Hermes-3; RE mapping -> 7 clones (5 of which covered the same region & were combined into one); subcloning into M13mp18, Sanger sequencing, PepPlot computer translation
Stamenkovic et al. 1989 [11]	U937, JY, Raji, KG-1 cells used to created cDNA libraries; cDNA then transfected into COS cells; screening with anti-CD44 Abs, immune-panning, washing, cell lysis, DNA purification, transformation into E. coli; two more rounds of enrichment; plasmid DNA recovery, does not mention sequencing method used, but presents sequences for 3 variant clones
Harn, et al. 1991 [64]	- Reticulocyte cDNA amplified by PCR; PCR product used in screening of reticulocyte CD44 in established CD44 cDNA libraries, chosen clones subcloned into plasmids and sequenced by Sanger method, sequence analysis performed with MacVector software
Shitvelman & Bishop, 1991 [52]	Full-length CD44 from human placental and melanoma cell line HT144 RNA cDNA libraries, subcloned into M13, Sanger sequenced
Stamenkovic, et al., 1991 [61]	I cDNA library from colon carcinoma HT29 cells transfected into COS cells; screened with anti-CD44 mAbs, immune-panned, adherent cells lysed, episomal DNA purified and transformed into E. coli; enrichment repeated twice; pDNA recovery from positive colonies and transfected to COS cells; RE and DNA sequenced
Dougherty, et al., 1991 [62]	RNA isolated from myelomonocytic KG1 cells using pCDw44 (sCD44 in CDM8 plasmid). Bacterial colonies used to transfect COS7 cells; cells screened with Abs for CD44; plasmid transformed back into bacteria, then again into COS7 and screened with anti-CD44 Abs. RE digestion, subcloning into pUC19, Sanger sequencing. CD44R1 and CD44R2 clone identified by PCR
Screaton, et al., 1992 [47]	YAC cDNA library screened for CD44, total yeast DNA transformed into E. coli, intron PCR products amplified and Sanger sequenced
Kugelman, et al., 1992 [60]	Keratinocyte cDNA library was transformed into E. coli, immuno-screened for CD44; positive clones were digested with RE and subcloned into pBluescript. Sanger sequencing was performed. Nucleotide sequence was determined for both strands from subcloned fragments and from nested sets of deletions (Henikoff method), or by using oligonucleotide primers prepared on the basis of known CD44 sequences
Gunther, 1993 [53]	Unpublished results, presumably Sanger sequencing (used in previous work)
Tanabe, et al., 1993 [57]	MCF-7, MDA1453, MDA468, A375, M151P, HL460A, HIT29, and SV40-transformed VA13 cells were cultured. CD44 cDNA was amplified via PCR (performed in triplicate). PCR product was cloned into pCR-1000 vector before performing Sanger sequencing
Chiu, et al., 1998 [58]	Source KG1a cells, sequencing method unknown
Venter, et al., 2001 [59]	Blood and semen samples from multiple donors (varying sex, ethnicity), preparation of plasmid libraries, Sanger sequencing, ABI PRISM 3700 DNA Analyzer sequence determination. Annotation of predicted CD44 isoform sequences by NCBI staff
Strausberg, et al., 2002 [54]	Primer walking, transposon insertions and concentrated shotgun sequencing of full-insert cDNA (full-ORF clones). Annotation of predicted CD44 isoform sequences by NCBI staff
Chr38p14	Based on Strausberg et al. 2002 & updates. To fill the remaining gaps a complete hydatidiform mole was sequenced by: 30x PacBio circular consensus (Hifi), 120x Oxford Nanopore ultralong-read, 100x Illumina PCR-Free, 70x Illumina Arima Genetics Hi-C Annotation of predicted CD44 isoform sequences by NCBI staff

Table S5. Chemical structures of small-molecule binders of CD44 from the available experimental structures.

PDB ID	Structure of the compound	PDB ID	Structure of the compound
	**Murine CD44 – X-ray structures**		
	**Liu & Finzel, 2014 [79]**		**Bradshaw, et al., 2021, unpublished [81]**
4MRD	HA4<br>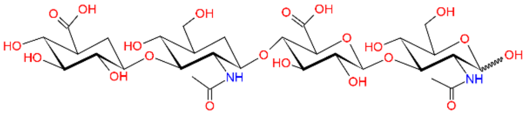	5SC0	Z2856434899: 1-[(thiophen-3-yl)methyl]piperidin-4-ol<br>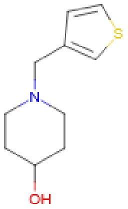
4MRE	3-methylbenzene-1,2-diamine<br>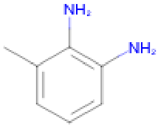	5SC1	Z431807512: N-methyl-3-oxo-N-(propan-2-yl)piperazine-1-sulfonamide<br>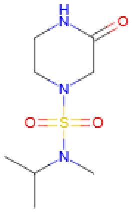
4MRF	1,2,3,4-tetrahydroisoquinoline<br>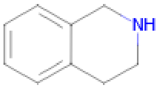	5SC2	Z190780124: (3S)-N-[(1R)-1-cyclopropylethyl]-2-oxo-2,3-dihydropyridine-3-carboxamide<br>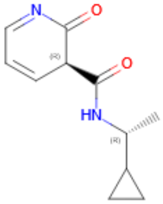
4MRG	1,2,3,4-tetrahydroisoquinolin-5-amine<br>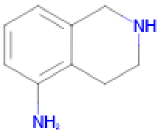	5SC3	Z422471910: N-[(1S)-1-cyclopropylethyl]-N,4-dimethyl-1,3-thiazole-5-carboxamide<br>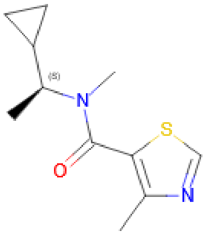
4MRH	4-chloro-5-methylbenzene-1,2-diamine<br>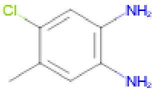	5SC4	Z927412236: (2R)-2-amino-1-[(2S)-2-methylpiperidin-1-yl]propan-1-one<br>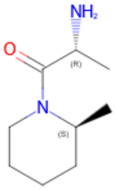

4NP2	2-[(4-methyl-1H-imidazol-5-yl)methyl]-1,2,3,4-tetrahydroisoquinoline 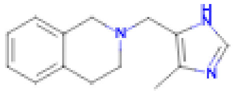	5SC5	Z56827661: N-(3-methylbenzene-1-carbonyl)glycine 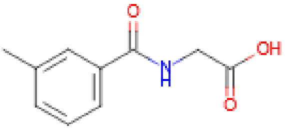
4NP3	2-[(4-methyl-1H-imidazol-5-yl)methyl]-1,2,3,4-tetrahydroisoquinolin-8-amine 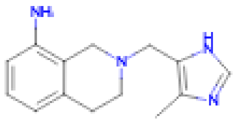	5SC6	POB0019: methyl (2S,3R)-1-(methanesulfonyl)-3-methylpiperidine-2-carboxylate 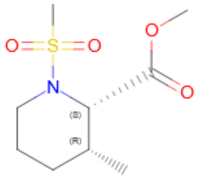
	**Liu & Finzel, 2016, unpublished [80]**	5SC7	POB0120: (2R)-1',4'-dihydro-2'H-spiro[pyrrolidine-2,3'-quinolin]-2'-one 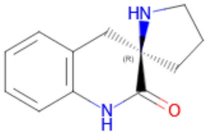
5BZC	4,4-dimethyl-1,2,3,4-tetrahydroisoquinoline 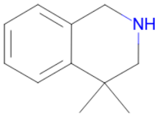		
5BZE	[(1R)-1,2,3,4-tetrahydroisoquinolin-1-yl]methanol / [(1S)-1,2,3,4-tetrahydroisoquinolin-1-yl]methanol 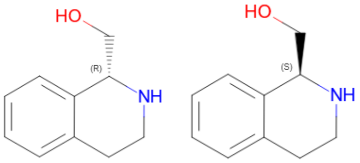	5SBK	Z1258992717: N-[(3R)-piperidin-3-yl]benzamide 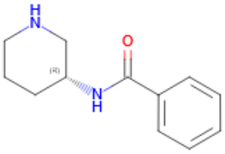
5BZF	2-ethyl-1,2,3,4-tetrahydroisoquinolin-5-amine 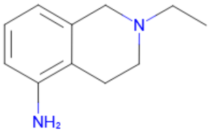	5SBL	Z126932614: 2-[(methylsulfonyl)methyl]-1H-benzimidazole 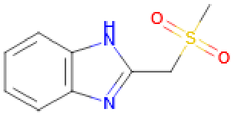
5BZG	2-methyl-1,2,3,4-tetrahydroisoquinolin-5-amine 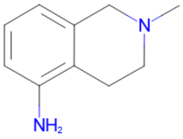	5SBM	Z1267885772: (3S)-3-methyl-1-(6-methylpyridin-2-yl)piperazine 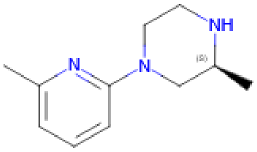
5BZH	3-(3,4-dihydroisoquinolin-2(1H)-yl)propan-1-amine 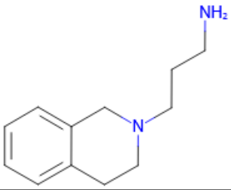	5SBN	Z57040482: N-(2-hydroxyphenyl)acetamide 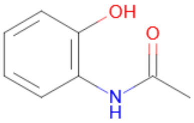

5BZI	1,2,3,4-tetrahydroisoquinolin-8-amine<br>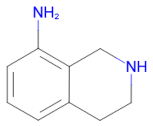	5SBO	Z2856434878: 4-[(3,4-dimethoxyphenyl)methyl]morpholine<br>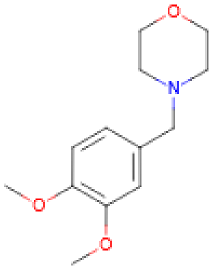
5BZJ	2-[2-(1-methyl-1H-imidazol-2-yl)ethyl]-1,2,3,4-tetrahydroisoquinolin-8-amine<br>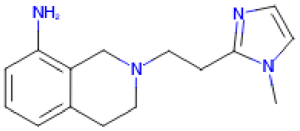	5SBP	Z1229798311: 2-acetamido-6-fluorobenzoic acid<br>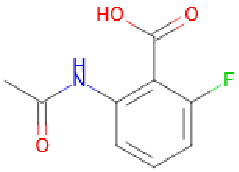
5BZK	methyl 3-(3,4-dihydroisoquinolin-2(1H)-yl)propanoate<br>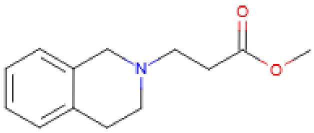	5SBQ	Z44592329: N-phenyl-N'-pyridin-3-ylurea<br>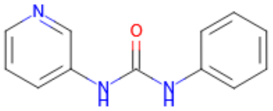
5BZL	methyl 3-(8-amino-3,4-dihydroisoquinolin-2(1H)-yl)propanoate<br>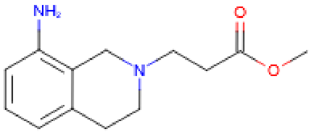	5SBR	Z1259341012: (1R)-1-[4-(morpholin-4-yl)phenyl]ethan-1-amine<br>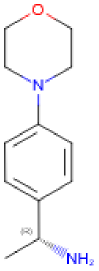
5BZM	2-[(4-amino-3-methylpyridin-2-yl)methyl]-1,2,3,4-tetrahydroisoquinolin-8-amine<br>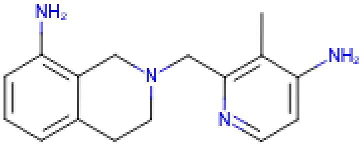	5SBS	Z340495298: N-cyclopropyl-5-methyl-N-(propan-2-yl)-1,2-oxazole-3-carboxamide<br>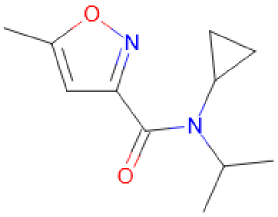

5BZN	2-(2-methoxyethyl)-1,2,3,4-tetrahydroisoquinolin-8-amine 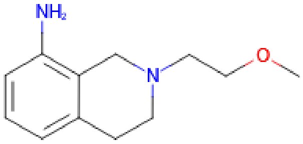	5SBT	Z445856640: N-[(3R)-1,1-dioxo-1lambda~6~-thiolan-3-yl]-N-methyl-N'-propan-2-ylurea 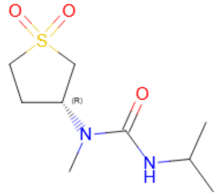
5BZO	3-(8-amino-3,4-dihydroisoquinolin-2(1H)-yl)-N-methylpropanamide 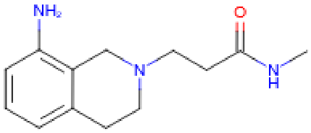	5SBU	Z839988838: N-[(3,5-dimethyl-1H-pyrazol-4-yl)methyl]cyclohexanamine 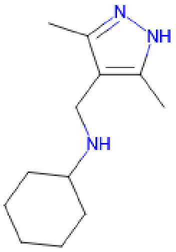
5BZP	2-[3-(dimethylamino)propyl]-1,2,3,4-tetrahydroisoquinolin-8-amine 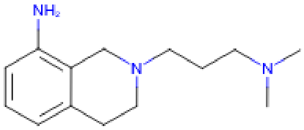	5SBV	Z31721798: 3-cyclohexyl-1-(morpholin-4-yl)propan-1-one 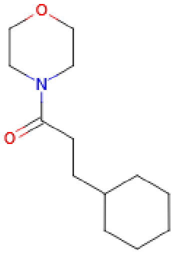
5BZQ	2-[3-(morpholin-4-yl)propyl]-1,2,3,4-tetrahydroisoquinolin-8-amine 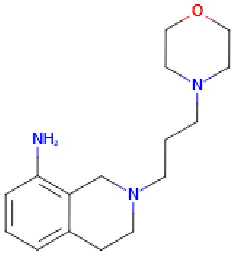	5SBW	Z2856434874: 1-{[4-(propan-2-yl)phenyl]methyl}piperidin-4-ol 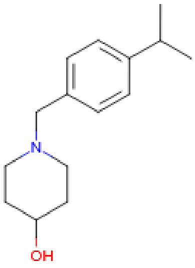
5BZR	2-[3-(tetrahydro-2H-pyran-4-yloxy)propyl]-1,2,3,4-tetrahydroisoquinolin-8-amine 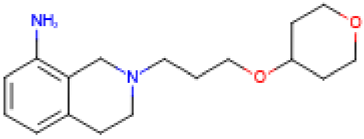	5SBX	Z53825479: N-cyclopentylmethanesulfonamide 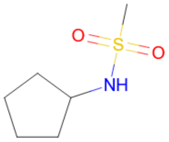

5BZS	2-[3-(tetrahydro-2H-pyran-4-yloxy)propyl]-1,2,3,4-tetrahydroisoquinolin-5-amine 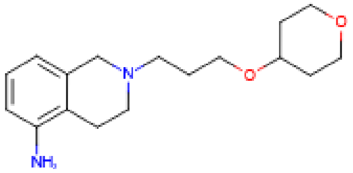	5SBY	Z1878656559: (1R,2R)-2,4,4-trimethyl-N-[(1H-pyrazol-3-yl)methyl]cyclopentan-1-amine 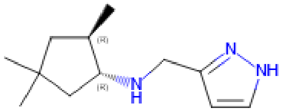
5BZT	2-(1,3-dimethoxypropan-2-yl)-1,2,3,4-tetrahydroisoquinolin-8-amine 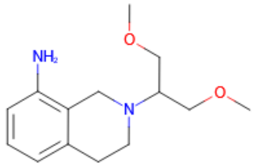	5SBZ	Z768399682: [(2R,5S)-2,5-dimethylmorpholin-4-yl](1,2,5-thiadiazol-3-yl)methanone 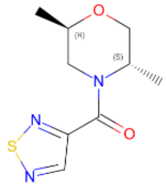
**Human CD44 – chosen compounds from the NMR binding studies**			
Baggio, et al., 2016 [85]	Compound 131B6 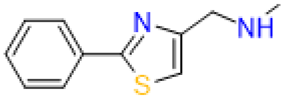		K_d = (7.43 ± 0.36) mM
Pustuła, et al., 2019 [86]	Compound 1 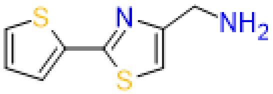		K_d = (0.895 ± 0.042) mM
	Compound 2 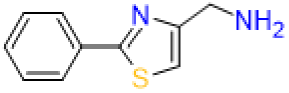		K_d = (2.56 ± 0.13) mM
	Compound 3 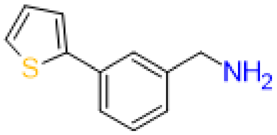		K_d = (0.662 ± 0.082) mM
